# Interpretation of ambiguous and unambiguous idiomatic statements in high and low schizotypal individuals

**DOI:** 10.1016/j.scog.2026.100461

**Published:** 2026-08-21

**Authors:** Lara Fernandes, Camille Dekeuleneer, Angélique Moroz, Luis Carlo Bulnes, Sandrine Detandt, Ariane Bazan

**Affiliations:** a Université de Lorraine, INTERPSY, F-54000 Nancy, France; b Université libre de Bruxelles, Faculté de Psychologie, Sciences de l'éducation et de logopédie (ULB), Belgium; c Parhélie — centre psychiatrique pour mineurs, Belgium; d Brain Body & Cognition Research Group, Vrije Universiteit Brussel, Belgium

**Keywords:** Schizotypy, Figurative language, Idioms, Concreteness, Literal bias, Schizophrenia continuum

## Abstract

Adopting a continuum perspective, this study examined figurative language comprehension in individuals with high or low levels of schizotypy within a non-clinical population. We investigated whether difficulties in figurative language processing are specifically driven by the presence of a competing literal interpretation. We hypothesized that higher schizotypy would be associated with fewer figurative responses and more literal and concrete responses for ambiguous idioms only, with no comparable effect for unambiguous idioms. A total of 377 French-speaking adults from the general population completed an idiom comprehension task and the Schizotypal Personality Questionnaire (SPQ). Based on total SPQ scores, 139 participants were included in the group analyses (73 high schizotypy, 66 low schizotypy). The task combined two methodological approaches previously examined separately by including both ambiguous and unambiguous idioms while distinguishing figurative, literal, and concrete response options. Participants with high schizotypy produced significantly fewer figurative responses and significantly more concrete responses than those with low schizotypy, whereas no difference was observed for literal responses. Importantly, these group differences were found regardless of idiom ambiguity, indicating that they were not specifically driven by the presence of a competing literal interpretation. These findings suggest that reduced figurative interpretations in schizotypy cannot be explained solely by competition from a plausible literal interpretation. More importantly, they highlight that distinguishing literal from concrete response options may help clarify previous inconsistencies in figurative language research in schizotypy and schizophrenia.

## Highlights

• Idiom comprehension was examined in high and low schizotypy.
• Ambiguity manipulation tested competition between figurative and literal meanings.
• High schizotypy produced fewer figurative interpretations.
• High schizotypy showed increased concrete interpretations.
• Findings support a continuum model with language impairments in schizophrenia.

## 1 Introduction

For several decades, numerous studies have shown that individuals with schizophrenia experience difficulties interpreting various forms of expression, including metaphors, idioms, proverbs, humor, and irony, with a recurrent tendency to favor literal over figurative interpretations (Haas et al., 2015; Mossaheb et al., 2014; Iakimova et al., 2006, Iakimova et al., 2010; Schettino et al., 2010; Titone et al., 2002; Adamczyk et al., 2017; Langdon et al., 2002). Beyond clinical schizophrenia, researchers have increasingly investigated schizotypy, a multidimensional personality construct reflecting subclinical expressions of schizophrenia-spectrum traits, distributed continuously in the general population (Raine, 1991; Ettinger et al., 2014). According to Raine's framework, this construct includes cognitive-perceptual, interpersonal, and disorganization dimensions (Raine, 1991; Raine et al., 1994; Gruzelier, 1995). Within continuum models, schizotypy provides a useful non-clinical framework for investigating cognitive mechanisms related to schizophrenia without confounds associated with psychosis, hospitalization, or medication (Del Goleto et al., 2016). Evidence from social cognition, cognitive, neurobiological, and genetic domains supports a partial overlap between schizotypy and schizophrenia (Del Goleto et al., 2016; Ettinger et al., 2014).

In the domain of figurative language, Langdon and Coltheart (2004) tested participants of a general population split at the median of the Schizotypal personality questionnaire (SPQ). Participants read short stories ending with a metaphorical statement and were asked to judge whether the statement made sense in context. Contrary to expectations, individuals with high schizotypy did not exhibit impairments in metaphor comprehension. However, these findings were later questioned as the nature of the metaphors used was considered largely lexicalized and thus the materials might not have tested the genuine metaphorizing capacities of the participants (Humphrey et al., 2010). Using novel non-lexicalized metaphors, Fernandes et al. (2025) showed that higher levels of schizotypy were indeed associated with difficulties in metaphor comprehension, reflected in an increased preference for literal and concrete interpretations. While some studies have suggested that figurative language difficulties may be more closely related to specific schizotypy dimensions, particularly interpersonal deficits (e.g., Langdon and Coltheart, 2004) or cognitive-perceptual (e.g., Humphrey et al., 2010), Fernandes et al. (2025) found contributions from all three SPQ dimensions.

Beyond metaphors, idioms constitute a particularly relevant domain for investigating contextual processing in schizophrenia, insofar as their figurative meaning emerges exclusively from a specific lexical configuration and cannot be inferred from the individual words alone (Titone et al., 2002). Idioms can also be classified according to their familiarity, predictability, and degree of ambiguity, depending on whether a plausible literal interpretation is possible (Sela et al., 2015).

According to the contextual integration deficit hypothesis (Chapman, 1960), schizophrenia patients make less efficient use of relevant contextual cues. For instance, when confronted with the expression “*His farewell letter contained daggers…*”, an interpretation such as “*And I removed them from the envelope*” illustrates a failure to establish a semantic link between *daggers* and *letters*, leaving only the dominant literal meaning of *dagger* activated. Other studies, by contrast, suggest that context is detected, but that difficulties in inhibition limit access to figurative meaning when a literal interpretation remains plausible (Titone et al., 2002). In line with this latter perspective, Titone et al. (2002) showed that patients with schizophrenia differed from controls only when idioms were semantically ambiguous and allowed a plausible literal interpretation (e.g., “*kicked the bucket*”), but not when the literal meaning was implausible (e.g., “*to be on cloud nine*”). These findings suggest that the primary difficulty lies not in idiom comprehension per se, but in inhibiting competing literal interpretations.

However, Iakimova et al. (2006) propose that earlier studies likely overestimated the literal bias, by failing to distinguish between two different non-figurative response options. Indeed, these authors highlight that most experimental protocols in previous research contrast only two types of responses — literal and figurative, while Iakimova et al. (2006) also distinguish concrete response options. Literalness refers to a coherent global interpretation based on the primary meaning of the expression, for example, interpreting “*The grass is always greener on the other side*” as “*The neighbor's garden is better maintained*” whereas concreteness reflects a focus on an isolated, often salient, element of the expression, without necessarily considering its overall meaning. For instance, interpreting the same idiom as “*The lawnmower is effective*”. Studies lacking this distinction might have biased literal response rates since any non-figurative response is automatically classified as literal.

Using this methodological refinement, Iakimova et al. (2006) examined ambiguous idiom comprehension in patients with schizophrenia and healthy controls through a multiple-choice paradigm distinguishing figurative, literal, and concrete responses. Despite a strong contextual bias favoring figurative interpretation, patients with schizophrenia produced significantly fewer figurative responses and showed a tendency to provide more literal and concrete responses than control participants. A parallel comparison with a control group of depressive patients, however, did not show this bias towards more literal responses in depressive patients, but did show a comparable increase in concrete responses. Accordingly, Iakimova et al. (2006) do not propose a single explanatory mechanism for their results but suggest that difficulties in accessing figurative meaning may result from a variety of cognitive constraints, including difficulties in semantic context processing. Despite this methodological advance, Iakimova et al. (2006) focused exclusively on ambiguous idioms. It therefore remains unclear whether the differences in figurative, literal and concrete responses persist across both ambiguous and unambiguous idioms, allowing us to isolate the potential effect of idiom ambiguity specifically on the response preference. Moreover, although we believe Iakimova et al.'s (2006) distinction between literal and concrete responses is crucial, the literal response options in that study included lexical cues that were highly associated, likely triggering concrete processing and resulting in an insufficient distinction between the two response categories.

### 1.1 The present study

The present study extends previous work in three ways. First, despite promising evidence supporting continuum models between schizotypy and schizophrenia, figurative language research in schizotypy remains limited. The present study therefore aims to extend this limited line of research, while taking advantage of a non-clinical schizotypy model that allows the inclusion of healthy adults and thus the recruitment of a larger sample. Second, we combine two methodological approaches that have previously been examined separately: the ambiguity manipulation used by Titone et al. (2002) and the distinction between figurative, literal, and concrete response types introduced by Iakimova et al. (2006). Third, we further refined the construction of response options to more strictly disentangle literal and concrete interpretations, by minimizing associative overlap between response categories.

Based on inhibition accounts of figurative language processing (Titone et al., 2002), we hypothesized that individuals with higher schizotypy would produce fewer figurative responses and more literal and concrete responses specifically for ambiguous idioms, where a competing literal interpretation is available, with no comparable effect expected for unambiguous idioms.

## 2 Method

### 2.1 Participants

Participants were recruited via the social media platform Facebook, where announcements and invitations to participate in the study were posted through personal accounts of colleagues and various public Facebook groups (e.g., residents of several towns, students from different universities, selected arbitrarily and distinct from our own). After applying the eligibility criteria (age ≥ 18 years, a minimum French proficiency score of 5 out of 7 on a self-rated language proficiency scale, where 0 indicated “no knowledge” and 7 indicated “native or native-like proficiency,” and complete data), the final sample comprised 377 participants. Written consent was obtained from all participants according to the protocol approved by the ethics committee of the Université libre de Bruxelles.

Participants ranged in age from 18 to 91 years (*M* = 40.0, *SD* = 16.5), and 77.3% were women. They had completed an average of 15.0 years of education (*SD* = 2.7). Self-rated French proficiency was high (*M* = 6.9 out of 7, *SD* = 0.3), and 55.7% reported being bilingual.

### 2.2 Procedure and materials

#### 2.2.1 Procedure

Participants completed an online questionnaire administered via LimeSurvey, autonomously and without intervention from the research team. Participants first completed demographic questions, followed by the *Idiom Comprehension Task* and then the *Schizotypal Personality Questionnaire*. The dataset supporting the findings of this study is available in the DOREL repository (Université de Lorraine).

### 2.3 Materials

#### 2.3.1 The schizotypal personality questionnaire

Schizotypal personality traits were assessed using the French-validated version of the Schizotypal Personality Questionnaire (SPQ; Raine, 1991), validated by Badoud et al. (2011). The SPQ is a 74-item self-report questionnaire with dichotomous (yes/no) response options based on DSM-III-R criteria for schizotypal personality disorder. It comprises nine subscales: ideas of reference, magical thinking, unusual perceptual experiences, suspiciousness, paranoia, social anxiety, eccentric behavior, disorganized speech, few close friends, and constricted affect.

To define the schizotypy groups used in the main analyses, participants were classified according to their total SPQ scores. Participants scoring one standard deviation below the mean (SPQ ≤ 11) were categorized as low-schizotypal (lo-S), while those scoring one standard deviation above the mean (SPQ ≥ 39) were categorized as high-schizotypal (hi-S). This resulted in a sample of 139 participants: 66 lo-S and 73 hi-S. Because no single grouping criterion has been universally adopted in the schizotypy literature, our grouping strategy was selected based on the characteristics of the present sample. Whereas previous studies have used median splits (Langdon and Coltheart, 2004), quartile-based selection (Humphrey et al., 2010), or more restrictive cut-offs (Raine, 1991), we adopted a ± 1 SD criterion because our study relied on a non-clinical adult community sample, consistent with recommendations from Chan et al. (2019).

Internal consistency was excellent for the total SPQ scale (α = 0.94) and for its three subscales (Cognitive-Perceptual: α = 0.87; Interpersonal: α = 0.91; Disorganization: α = 0.86)*.*

#### 2.3.2 The idiom comprehension task

The Idiom Comprehension Task consisted of twenty French idiomatic expressions composed of three to five words. Ten idioms were literally plausible (ambiguous idioms), whereas ten were literally implausible (unambiguous idioms) (see Appendix A).

#### 2.3.3 Ambiguous idioms

We used the ten ambiguous idioms (in French) from Iakimova et al.'s (2006) study, originally selected from the IDIOMATIC database (Denhière and Verstiggel, 1998). Additional details regarding the selection of ambiguous idioms are provided in the Supplementary Methods.

Together with the ten selected idiomatic expressions, Iakimova et al. (2006) proposed four keywords representing figurative, literal, concrete, and random interpretations.

In the present study, rather than presenting isolated keywords, each idiom was embedded in a neutral sentence context. For example, the French idiom “*Pendant le match*, *notre équipe a ramassé une veste…*” which means, in English, “During the match, our team took a beating” in the figurative meaning and “During the match, our team picked up a coat…” in the literal meaning. Participants then selected among three sentence completions corresponding to figurative, literal, or concrete interpretations; the random option proposed by Iakimova et al. (2006) was not included. The response alternatives were constructed by expanding the keywords into short sentence continuations: figurative (e.g., *… et ils ont échoué lamentablement* / “… and they failed miserably”), literal (e.g., *… et ils l'ont prise en partant* / “… and they took it with them when they left”), and concrete (e.g., *… et les cravates sont élégantes*/“… and ties are elegant”; Table 1).

**Table 1:** Ambiguous idiomatic expressions selected from Iakimova et al. (2006) and their modifications in the present study (last column).

Idiom (English translation)	Figurative	Literal	Concrete	Modifications
Régler son compte (Settle one's account)	se venger (revenge)	**effacer** (erase)	banque (bank)	Replaced « rembourser » (reimburse) because « rembourser » is too strongly associated with “régler” (settle).
Tomber du ciel (Fall from the sky)	inespéré (unexpected)	grêle (hail)	azur (blue)	Unchanged
Mettre le doigt dans l'engrenage (Put your finger in the gears)	imprudence (imprudence)	se blesser (be injured)	machine (machine)	Unchanged
Couper le cordon (Cut the cord)	séparer (separate)	**outil** (tool)	fil (string)	Replaced « ciseaux » (scissors) because « ciseaux » is too strongly associated with “couper” (cut).
Ramasser une veste (Pick up a coat)	échouer (fail)	prendre (take)	cravate (tie)	Unchanged
Tourner en rond (Turn in circles)	désoeuvré (disoriented)	toupie (spinning top)	cercle (circle)	Unchanged
Montrer les dents (Show one's teeth)	menacer (threaten)	**molosse** (mastiff)	carie (caries)	Replaced « chien » (dog) because « chien » is highly associated with “dents” (teeth).
Avaler la pilule (Swallow the pill)	accepter (accept)	**arthrite** (arthritis)	comprimé (tablet)	Replaced « malade » (ill) because « malade » is too strongly associated with “pilule” (pill).
Mettre la main au feu (Put one's hand in the fire)	certain (sure)	maladroit (clumsy)	flamme (flame)	Unchanged
Laisser une ardoise (Leave a slate/unpaid bill)	dû (debt)	couvreur (roofer)	craie (chalk)	Unchanged

Note. Idioms and response options are presented in French, with English translations in parentheses. Response options correspond to figurative, literal, and concrete response categories. Boldface indicates response options that were modified from the original idiom task by Iakimova et al. (2006). The rationale for each modification is provided in the Modifications column.

#### 2.3.4 Unambiguous idioms

Unambiguous idioms, by contrast, had only one possible figurative meaning and no viable literal interpretation. A typical example is *broyer du noir* (“grind blackness”), which can only be understood figuratively (“to feel down”). Ten unambiguous idioms were selected from Caillies (2009),^1^ a normative database of French idiomatic expressions including familiarity and literal plausibility ratings.

However, since no explicit thresholds were defined by the author, we applied the operational criteria used in previous studies by Iakimova et al. (2006) and Titone et al. (2002). Additional details regarding these selection criteria are provided in the Supplementary methods.

As no response options were provided in the literature, three response alternatives (figurative, literal and concrete) were developed for each idiom. Figurative responses reflected the conventional idiomatic meaning, literal responses were derived by decomposing the expression into its lexical components and combining their primary dictionary meanings, and concrete responses were based on French associative norms, selecting the word that was semantically or associatively most closely related to the idiom's central element. See Supplementary Methods for additional details and examples of the response construction procedure.

Once selected, these words were integrated into short, grammatically coherent sentence completions, adhering to the same syntactic and semantic constraints used for the ambiguous idioms (Table 2).

**Table 2:** Unambiguous idioms selected from Caillies (2009).

Idiom	Figurative	Literal	Concrete
Bourrer le crâne (Stuff one's skull)	convaincre (convince)	lourd (heavy)	os (bones)
Broyer du noir (Grind blackness)	désespéré (desperate)	poudre (powder)	sombre (dark)
Brûler les étapes (Burn steps)	précipité (hasty)	escaliers (stairs)	incendie (fire)
Se la couler douce (Let it flow sweetly)	détente (relaxation)	consistance (consistency)	liquide (liquid)
Donner sa langue au chat (Give one's tongue to the cat)	abandonner (give up)	voulu (wanted)	chien (dog)
Demander la lune (Ask for the moon)	irréaliste (unrealistic)	distance (distance)	nuit (night)
Etre aux anges (Be with the angels)	enchantée (delighted)	quitter (leave)	ailes (wings)
Jeter un froid (Throw a chill)	malaise (awkwardness)	terre (ground)	neige (snow)
Tuer le temps (Kill time)	occupée (busy)	exécution (execution)	meurtre (murder)
Perdre la tête (Lose one's mind)	affolé (panicked)	localiser (locate)	cerveau (brain)

Note. Idioms and response options are presented in French, with English translations in parentheses. Response options correspond to figurative, literal, and concrete response categories.

#### 2.3.5 Construction principles for response options across both ambiguous and unambiguous idioms

The construction of sentence contexts and response options followed strict linguistic and associative constraints. First, literal responses could not also be concrete, in order to disentangle these two response modalities. Literal responses were intended to reflect the global literal meaning of the expression, whereas concrete responses relied on a salient associative element of one word within the idiom. If a literal response contains a highly associated concrete word, it becomes impossible to determine whether the response was chosen because of the global literal interpretation or because of the salient associative cue. For example, in « En déménageant, Arthur a décidé de couper le cordon… » (“By moving out, Arthur decided to cut the cord…”), the literal completion « … et pour cela, il a utilisé des ciseaux. » (“… and for that, he used scissors.”) would combine a global literal interpretation with a strong associative link (couper–ciseau). We therefore did not retain the suggestion of Iakimova et al. (2006) (“ciseaux”) and replaced it with « … et pour cela, il a utilisé un outil. » (“… and to do so, he used a tool.”), where the association between *couper* and *outil* is much weaker.

Second, concrete responses could not also be literal, since concreteness refers to a surface-level, word-based processing that ignores sentence meaning. For example, in *« Il a mis le doigt dans l'engrenage… »* (“He got his finger caught in the gears…”), the response *« …et la machine a directement été coupée »* (“...and the machine immediately stopped”) would not be allowed, because it implies a coherent integration of the sentence context while also containing a word (*machine*) associated with the root word (*engrenage*).

#### 2.3.6 Reliability

The Idioms Comprehension Task demonstrated good internal consistency. Reliability analyses yielded a Cronbach's alpha of 0.86 for ambiguous idioms, 0.76 for unambiguous idioms, and 0.89 for the entire set of twenty idioms when considered together.

### 2.4 Study design and data analysis

The study employed a 2 × 2 × 3 mixed design, with schizotypy group (high vs. low) as a between-subjects factor, and idiom ambiguity (ambiguous vs. unambiguous) and response type (figurative, literal, concrete) as within-subject factors. The dependent variable was the number of responses selected for each response type in each ambiguity condition.

An a priori power analysis was conducted using G*Power to estimate the sample size required to detect a medium effect size (f = 0.25; Cohen, 1988) in a repeated-measures ANOVA including within- and between-subjects factors. The design comprised two groups (hi-S vs. lo-S) and six within-subject conditions (three response types × two ambiguity levels). With α = 0.05, power (1 − β) = 0.80, an assumed correlation of 0.50 between measures, and a nonsphericity correction ε = 0.70, the required total sample size was 24 participants. The final sample comprised 139 participants (66 lo-S, 73 hi-S).

To characterize the sample, associations between demographic variables (age, gender, and education) and continuous SPQ scores were examined in the full sample using Pearson correlation analyses.

To assess demographic comparability between the high- and low-schizotypy groups, the groups were compared on age and education using Mann–Whitney *U* tests and on gender distribution using a chi-square test.

The main hypothesis was tested using a repeated-measures ANOVA corresponding to the mixed design described above. Greenhouse–Geisser corrections were applied when the assumption of sphericity was violated.

Where significant interactions emerged in the ANOVA, planned follow-up comparisons were conducted to clarify the direction of the observed effects. Because several dependent variables violated the assumption of normality, Wilcoxon signed-rank tests were used for within-group pairwise comparisons.

Additional regression analyses were conducted to examine (1) the effects of age and gender on figurative, literal, and concrete responses within the low and high schizotypy groups, and (2) the unique contributions of the three SPQ dimensions to each response type across the full sample. These supplementary analyses are reported in the Supplementary Results.

## 3 Results

### 3.1 Demographic and group characteristics

#### 3.1.1 Schizotypy characteristics

Total SPQ scores ranged from 0 to 64 with *M* = 24.9 (*SD* = 14.1), with *M* = 6.9, 9.4 and 5.9 for the Cognitive-perceptual (on 25), Interpersonal (on 33) and Disorganization (on 16) subscales respectively.

#### 3.1.2 Associations between demographic variables and SPQ

Across the full sample, gender was not associated with SPQ scores (*r* = −0.014, *p* = .393, 95% CI [−0.16, 0.18]). SPQ scores decreased with increasing age (*r* = −0.158, *p* < .001, 95% CI [−0.29, −0.10]) and with increasing years of education (*r* = −0.124; *p* < .001, 95% CI [−0.32, −0.12]).

#### 3.1.3 Group comparability

Comparisons between the hi-S and lo-S groups revealed that the hi-S group is significantly younger than the lo-S group (U = 1675.5, *p* = .002, *r* = 0.26, 95% CI [0.10, 0.41]). However, gender distribution did not significantly differ between the hi-S and lo-S groups (*χ*^*2*^(1) = 0.43, *p* = .513).

The hi-S group scored significantly higher than the lo-S group on all SPQ subscales: Disorganization (U = 17.0, *p* < .001, *r* = 0.52, 95% CI [0.39, 0.63]), Cognitive-Perceptual (U = 35.5, *p* < .001, *r* = 0.59, 95% CI [0.47, 0.69]), and Interpersonal (U = 4.0, *p* < .001, r = 0.52, 95% CI [0.39, 0.63]) (Table 3).

**Table 3:** Descriptive statistics and group comparisons for demographic variables, SPQ Scores, and Idiom comprehension task.

	Hi-S (N = 73)		Lo-S (N = 66)		*r*
	*M*	*SD*	*M*	*SD*	
Demography					
Age	36.4*	15.2	44.6	16.5	0.26
Education	13.2*	2.8	15.6	2.6	0.44
Total SPQ	47.7*	6.2	6.9	2.8	
Idioms comprehension task					
Ambiguous idioms					
Figurative	8.2*	1.9	8.8	1.5	0.17
Literal	1.4	1.3	1.1	1.2	0.15
Concrete	0.4	1.0	0.1	0.5	0.16
Unambiguous idioms					
Figurative	8.6*	2.2	9.2	1.7	0.25
Literal	1.0*	1.5	0.6	1.3	0.25
Concrete	0.4	0.9	0.2	0.5	0.13

Note. Values in the Hi-S and Lo-S columns represent group means (*M*) and standard deviations (*SD*). SPQ = Schizotypal Personality Questionnaire; Hi-S = high schizotypy group; Lo-S = low schizotypy group. Age and education are reported in years. SPQ total scores range from 0 to 74. For the Idiom comprehension task, values represent the mean number of responses selected by participants in each group for each response category within each idiom type, out of 10 idioms. The *r* values represent effect sizes. Asterisks indicate statistically significant between-group differences at *p* < .05.

To examine the potential influence of demographic variables on idiom interpretation within the two schizotypy groups, additional linear regression analyses were conducted separately in the high and low schizotypy groups (Supplementary Tables S1 and S2).

### 3.2 Main analysis

To test the main hypothesis, the planned repeated-measures ANOVA was conducted.

A significant main effect of response type was observed, *F*(1.14,155.47) = 1280.06, *p* < .001, η^2^ₚ = 0.903, indicating that the mean number of responses selected differed across the three response types. On average, figurative responses were the most frequent, followed by literal responses, and finally concrete responses (*M* = 8.5 > 1.2 > 0.3). No main effects of ambiguity condition or group were observed (*p* = 1).

**Fig. 1:**
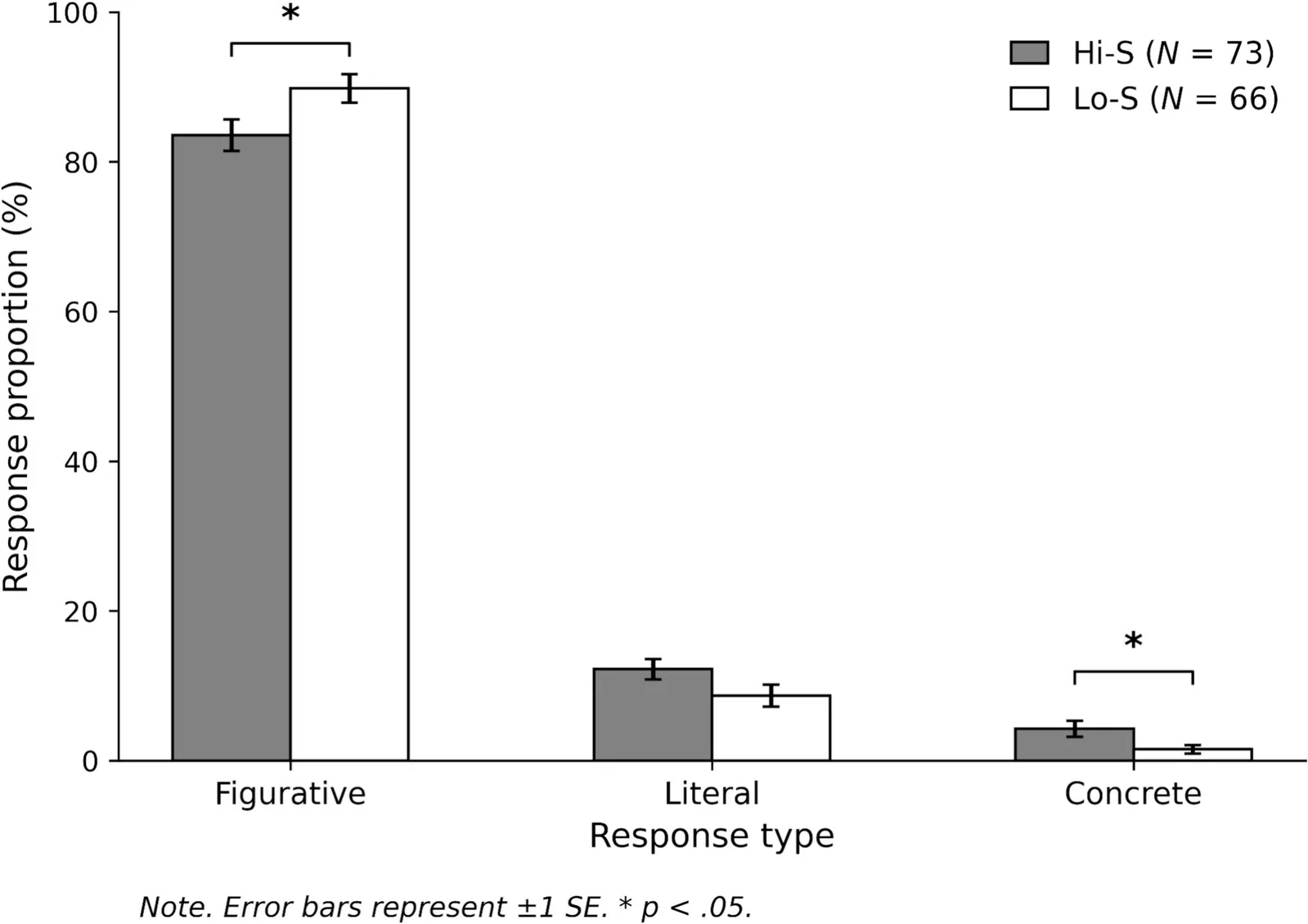
Response proportions (%) for figurative, literal and concrete responses on the sum of all idioms, both ambiguous and non-ambiguous, in high and low schizotypal participants.

The interaction between response type and schizotypy group was significant, *F*(1.14,155.47) = 4.36, *p* = .034, η^2^ₚ = 0.031 (see Table 4). Simple effects analyses showed that participants in the hi-S group produced significantly fewer figurative responses than those in the lo-S group, *F*(1,137) = 4.80, *p* = .030, and significantly more concrete responses, *F*(1,137) = 5.43, *p* = .021. No significant group difference was found for literal responses, *F*(1,137) = 3.09, *p* = .081 (see Fig. 1).

**Table 4:** Means of figurative, literal, and concrete responses across all idioms by schizotypy group.

	Figurative		Literal		Concrete	
	*M*	*SD*	*M*	*SD*	*M*	*SD*
Hi-S	16.7⁎	3.6	2.4	2.3	0.9⁎	1.8
Lo-S	18	3.1	1.7	2.4	0.3	0.9

Note. Values represent the mean number of figurative, literal, and concrete responses selected across ambiguous and unambiguous idioms, out of 20 idioms. *M* = mean; *SD* = standard deviation; Hi-S = high schizotypy group; Lo-S = low schizotypy group.

⁎ *p* < .05.

A significant interaction between ambiguity condition and response type was also found, *F*(1.28,175.93) = 11.97, *p* < .001, η^2^ₚ = 0.080. Mean comparisons indicated that ambiguous idioms elicited a lower proportion of figurative responses and a higher proportion of literal responses than unambiguous idioms. No significant difference was found between ambiguity conditions for concrete responses, *F*(1,137) = 0.58, *p* = .449.

Finally, the three-way interaction between the schizotypy group, ambiguity condition, and response type was not significant, *F*(1.28,175.93) = 0.12, *p* = .791, indicating that group and ambiguity did not jointly affect response type.

### 3.3 Follow-up comparisons

To further examine the significant interaction between ambiguity condition and response type, planned follow-up analyses were conducted using Wilcoxon signed-rank tests separately within each schizotypy group.

In the lo-S group, a significant effect of ambiguity was observed for literal responses (*z* = −3.873, *p* < .001) and for figurative responses (*z* = −3.276, *p* = .001). No significant difference was found for concrete responses (*z* = −0.973, *p* = .331).

In the hi-S group, ambiguity significantly affected literal responses (*z* = 2.257, *p* = .021) and tended to affect figurative responses (*z* = −1.913, *p* = .056). No significant difference was found for concrete responses (*z* = −0.227, *p* = .821).

## 4 Discussion and conclusion

The present study aimed to examine idiomatic language comprehension in individuals with high levels of schizotypy, focusing on figurative, literal, and concrete interpretations as a function of idiom ambiguity. It was hypothesized that difficulties in accessing figurative meaning would emerge exclusively for ambiguous idioms. A main effect of response type was observed: figurative responses were by far the most frequent, followed by literal responses and then concrete responses (8.5 vs. 1.2 vs. 0.3 out of 10). This distribution confirms that the task elicited the expected response hierarchy and that this non-clinical population generally understood the idiomatic nature of the expressions presented. These results are comparable to those reported by Iakimova et al. (2006) in control participants (89.3% figurative, 9.3% literal and 1.4% concrete responses).

The significant interaction between response type and SPQ group indicates that hi-S participants produced fewer figurative responses and more concrete responses than lo-S participants, independently of idiom ambiguity. A trend-level difference was also observed for literal responses. These findings highlight the importance of including both literal and concrete response options when investigating figurative language comprehension. As shown by Iakimova et al. (2006), a binary figurative–literal approach may obscure distinct interpretative profiles by conflating literalness and concreteness. To address this methodological limitation, Iakimova et al. (2006) introduced concreteness as an additional response category.

Converging results from Iakimova et al. (2006) in patients with schizophrenia and from Fernandes et al. (2025) in individuals with high levels of schizotypy further support this distinction. In both studies, participants produced more literal (20.0% in patients with schizophrenia and 20.0% in hi-S participants) and concrete responses (11.4% in patients with schizophrenia and 7.2% in hi-S participants) than the control group. Similarly, in the present study, hi-S participants selected both literal (12.2%) and concrete responses (4.0%). However, the present study revealed a significant difference between hi-S and lo-S participants specifically in the proportion of concrete responses, which were more frequent in the hi-S group, whereas the difference in literal responses reached only a trend level. In Iakimova et al. (2006), concrete responses (11.4% in patients with schizophrenia) were also more frequent than in controls but did not specifically distinguish patients with schizophrenia from patients with depression.

The interaction between idiom ambiguity and response type showed that ambiguous idioms elicited fewer figurative responses and more literal responses than non-ambiguous idioms, while no difference was observed for concrete responses. In other words, when idioms allow both a figurative and a literal interpretation, participants tend to favor the literal meaning more than when no literal interpretation is available. This pattern of results supports the validity of the ambiguity manipulation and provides additional evidence for the construct validity of the task, confirming that the ambiguity manipulation successfully induced the expected competition between figurative and literal interpretations.

However, this pattern was observed similarly in participants with lo and hi-S scores: the absence of a three-way interaction indicates that the reduced production of figurative interpretations observed in hi-S participants does not depend on idiom ambiguity. This difficulty therefore does not manifest specifically in contexts where a competing literal interpretation is available. Therefore our results differ from those of Titone et al. (2002), who reported reduced figurative priming in schizophrenia only for literally plausible idioms. These findings were interpreted in terms of a difficulty inhibiting competing literal meanings. We suggest that the study by Titone et al. (2002) did not control certain critical parameters, such as, in the construction of the ambiguous idioms, the relative salience of idiomatic and literal interpretations. In contrast to Iakimova et al. (2006), the present study found only a non-significant trend for literal responses, whereas concrete responses were significantly more frequent in hi-S than in lo-S participants. However, Iakimova et al. (2006) did control for this relative salience in the construction of the ambiguous idioms with a mean probability of a literal interpretation of 62%, and of 46% for the mean probability a figurative interpretation, showing a balanced salience for both readings. Therefore, this methodological point may not be the reason for our different results.

The discrepancies of our results with those of Titone et al. (2002) and Iakimova et al. (2006) may reflect a difference between a clinical population of patients with schizophrenia and a non-clinical population of hi-S participants. However, we propose that other, methodological biases, should be clarified before considering this explanation. Indeed, we propose that in both studies, the respectively absent, or imperfect, distinction between literal and concrete responses might be an important source of bias. In the present study, particular care was taken to construct literal and concrete response options not only according to the criteria proposed by Iakimova et al. (2006), but also to ensure that literal options did not contain highly associative words linked to the prime sentence, in order to neutralize their concreteness. While in Titone et al. (2002) concrete response options were not proposed, we suggest that in Iakimova et al. (2006), some literal response options may have included salient concrete words – for example, the use of the word *scissors* in the literal option for the idiom *cut the cord* – thereby rendering the literal option concrete as well. Such highly associative words may provide an unfair concreteness advantage to literal response options. Thus, we propose that both in Titone et al. (2002) and in Iakimova et al. (2006) the ratio of literal responses may be artificially overestimated. Future studies should more carefully disentangle literalness and concreteness, particularly by avoiding highly associated words in literal responses. Importantly, once literal and concrete response options were more carefully dissociated, only concrete responses significantly differentiated the hi-S and lo-S groups, whereas literal responses showed only a trend-level difference. Taken together, the central difficulty that may characterize access to figurative interpretations of idiomatic language, in both schizophrenia and schizotypy, is the attentional challenge posed by the presence of highly associatively salient alternatives to the figurative expression. The present results do not exclude the possibility that the semantic context processing of the figurative option might be intact at first, and is then overridden by a difficult-to-inhibit salient element in another response option. Thus, in accordance with others, our results still suggest an inhibition difficulty in schizotypy/schizophrenia.

Exploratory analyses (Table S3) further suggested that, within the multidimensional structure of schizotypy, only the disorganization dimension showed unique associations with figurative, literal, and concrete responses. These findings should not be interpreted as inconsistent with the main results, as they reflect the unique contribution of individual schizotypy dimensions rather than differences between participants classified into high and low schizotypy groups. Although statistically significant, these dimensional effects were small and therefore warrant cautious interpretation.

Several limitations should be acknowledged. The unequal gender distribution in the present sample limited the possibility of reliably examining potential gender effects. The gender imbalance in volunteer-based research, particularly the overrepresentation of women, is a well-documented limitation in psychological research (Gosling et al., 2004; Borg et al., 2024). Future studies should aim to recruit more gender-balanced samples by implementing targeted recruitment strategies to improve the participation of underrepresented groups (Borg et al., 2024). An additional limitation concerns the age difference observed between the hi-S and lo-S groups. This reflects an inherent limitation of assessing schizotypy with the Schizotypal Personality Questionnaire (SPQ), as SPQ scores are known to decrease with age (e.g., Fossati et al., 2003; Badcock and Dragović, 2006). Although idiom salience was controlled, the exclusive use of figurative expressions in the multiple-choice questionnaire may have induced a contextual bias favoring figurative interpretations across participants (Iakimova et al., 2006). Given the relatively long duration of the assessment (c. 45 min), fatigue may have affected performance. Importantly, the study was limited to the analysis of response proportions and did not include temporal measures (e.g., response latencies). Future studies incorporating temporal analyses could provide further insight into the stages involved in accessing literal and figurative meanings of ambiguous and non-ambiguous idioms. In addition, schizotypy was examined using a grouping approach rather than being analyzed as a continuous variable across the full spectrum of SPQ scores.

In conclusion, the present study extends previous work on figurative language comprehension in schizotypy by showing that reduced access to figurative interpretations is not specifically driven by idiom ambiguity. More importantly, our findings emphasize that carefully distinguishing literal and concrete response options may help clarify previous inconsistencies regarding figurative language comprehension in schizotypy and schizophrenia.

## CRediT authorship contribution statement

**Lara Fernandes:** Writing – review & editing, Writing – original draft, Visualization, Project administration, Methodology, Formal analysis, Data curation, Conceptualization. **Camille Dekeuleneer:** Visualization, Methodology, Investigation, Data curation. **Angélique Moroz:** Visualization, Methodology, Investigation, Data curation. **Luis Carlo Bulnes:** Writing – review & editing, Formal analysis. **Sandrine Detandt:** Writing – review & editing, Validation, Supervision. **Ariane Bazan:** Writing – review & editing, Writing – original draft, Validation, Supervision, Conceptualization, Metholodology.

## Declaration of competing interest

The authors declare that they have no known competing financial interests or personal relationships that could have appeared to influence the work reported in this paper.

## Footnotes

1 We did not use the IDIOMATIC database developed by Denhière and Verstiggel (1998) because we did not have access.
