# Supplementary material for: Interpretation of ambiguous and unambiguous idiomatic statements in high and low schizotypal individuals
Source: Schizophr Res Cogn. 2026 Aug 21;46:100461. doi: 10.1016/j.scog.2026.100461 (PMC13524631; doi:10.1016/j.scog.2026.100461)
Supplement: Supplementary file 1 [file mmc1.docx]

### Appendix A Supplementary Data

## Idioms Comprehension Task (translation^[1]^)

**Instructions**

We will now present you with a series of triads made up of sentences. On each trial, one sentence followed by three options will be presented. You will need to choose which of the three options best completes the proposed sentence, according to you. There are no right or wrong answers. Please try to respond spontaneously*.*

Ambiguous idioms

Idiom 1

« He wrote a song to settle his account… »

- **Figurative:** and his revenge was a success.
- **Literal:** and his deficit was successfully erased.
- **Concrete:** and the bank makes an attractive offer.

Idiom 2

« This sudden financial support fell from the sky… »

- **Figurative:** and this good news was completely unexpected.
- **Literal:** and it is accompanied by hail.
- **Concrete:** and the color blue is magnificent.

Idiom 3

« By accepting this job, he put his finger in the gears… »

- **Figurative:** and this choice was imprudent.
- **Literal:** and he injured a limb.
- **Concrete:** and the machines are efficient.

Idiom 4

« By moving out, Arthur decided to cut the cord… »

- **Figurative:** and he managed to separate himself from his family.
- **Literal:** and to do so, he used a tool.
- **Concrete:** and the computer string is too long.

Idiom 5

« During the match, our team picked up a coat… »

- **Figurative:** and they failed miserably.
- **Literal:** and they took it home with them afterward.
- **Concrete:** and ties are elegant.

Idiom 6

« He spent a long time turning in circles… »

- **Figurative:** because he was disoriented.
- **Literal:** and it is truly a high-performance spinning top.
- **Concrete:** and the circle is drawn in pencil.

Idiom 7

« Facing his competitor, he showed his teeth… »

- **Figurative:** and threatened him with impressive innovations.
- **Literal:** and we saw that it was a truly fearsome mastiff.
- **Concrete:** and caries cause infections.

Idiom 8

« Despite her disappointment, she swallowed the pill… »

- **Figurative:** and she accepted the situation.
- **Literal:** to recover from her arthritis.
- **Concrete:** and the tablets are pink.

Idiom 9

« Carl is ready to put his hand in the fire… »

- **Figurative:** to prove that he is sure of what he claims.
- **Literal:** because he is a clumsy person.
- **Concrete:** and the flames reach the roof.

Idiom 10

« Marc didn’t pay for his coffee; he left a slate… »

- **Figurative:** and the waiter has cleared his debt.
- **Literal:** and the roofer repaired the attic.
- **Concrete:** and the chalk draws white lines.

Non ambiguous idioms

Idiom 1

«  His persuasive speech managed to stuff his skull … »

- **Figurative** : and he was conviced.
- **Literal** : and now he is very heavy.
- **Concrete** : and the bones are bright white.

Idiom 2

« After his breakup, he spent his days grinding blackness … »

- **Figurative** : and he seemed truly desperate.
- **Literal** : and there was only powder left.
- **Concrete** : and it’s a very dark tone.

Idiom 3

« To succeed faster, he burnt steps… »

- **Figurative :** but his approach was too hasty.
- **Literal** : and of the stairs, nothing remained.
- **Concrete** : and fires are terrible catastrophes.

Idiom 4

« During vacation, he likes to let it flow sweetly… »

- **Figurative** : by enjoying every moment of relaxation.
- **Literal** : but he quickly lost all his consistency.
- **Concrete** : and the liquid spreads quickly.

Idiom 5

« In the guessing game, she had to give her tongue to the cat… »

- **Figurative** : and she gave up the game.
- **Literal** : but he did not want it.
- **Concrete** : and dogs are very friendly.

Idiom 6

« His manager tends to ask him for the moon … »

- **Figurative** : and he has unrealistic demands.
- **Literal** : but it is located 384,400 km away.
- **Concrete** : and the night is silent.

Idiom 7

« When she learned the news, she was with the angels… »

- **Figurative** : and she had a delighted look.
- **Literal** : and she had to leave them hastily.
- **Concrete** : and the wings are large in size.

Idiom 8

**«** Marc’s inappropriate comment threw a cold… »

- **Figurative** : and created a general awkwardness.
- **Literal** : and now he is on the ground.
- **Concrete** : and the snow covers the landscape.

Idiom 9

« While waiting for the appointment, she reads a book to kill time… »

- **Figurative** : and she is busy all morning.
- **Literal** : and she finds a good method of execution.
- **Concrete** : and murders are frequent.

Idiom 10

« Faced with so much stress, he ended up losing his mind… »

- **Figurative** : and panicked in an unmanageable way.
- **Literal** : and now he can no longer locate it.
- **Concrete** : and the brain is a fascinating machine.

## Outil Idiomes (version originale^[2]^)

**Consigne**

Nous allons maintenant vous proposer une série de triades composées de phrases. A chaque essai, une phrase suivie de trois options vous seront présentés. Il faudra choisir laquelle des trois options complète le mieux la phrase proposée selon vous. Il n’y a ni bonnes ni mauvaises réponses. Merci de rester spontané.e.

Les idiomes ambigus

Idiome 1

« Il a écrit une chanson pour régler ses comptes... »

- **Figuratif :** et sa vengeance est une réussite.
- **Littérale :** et son déficit a bien été effacé.
- **Concrétude :** et la banque fait une offre intéressante.

Idiome 2

« Ce soutien financier inattendu tombe du ciel... »

- **Figuratif :** et cette bonne nouvelle était inespérée.
- **Littérale :** et il est accompagné́ de grêle.
- **Concrétude :** et la couleur azur est magnifique.

Idiome 3

« En acceptant ce job, il a mis le doigt dans l'engrenage... »

- **Figuratif :** et ce choix était imprudent.
- **Littérale :** et il s’est blessé un membre.
- **Concrétude :** et les machines sont performantes.

Idiome 4

« En déménageant, Arthur a décidé de couper le cordon... »

- **Figuratif :** et il est parvenu à se séparer de sa famille.
- **Littérale :** et pour cela, il a utilisé́ un outil.
- **Concrétude :** et le fil de l’ordinateur est trop long.

Idiome 5

« Lors du match, notre équipe a ramassé une veste... »

- **Figuratif :** et ils ont échoué lamentablement.
- **Littérale :** et ils l’ont prise avec eux en rentrant.
- **Concrétude :** et les cravates sont élégantes.

Idiome 6

« Il a passé́ longtemps à tourner en rond... »

- **Figuratif :** puisqu’il était désœuvré.
- **Littérale :** et c’est vraiment une toupie performante.
- **Concrétude :** et le cercle est dessiné au crayon.

Idiome 7

« Face à son concurrent, il a montré ses dents... »

- **Figuratif :** et le menace avec des innovations imposantes.
- **Littérale :** et nous avons vu que c’était un molosse vraiment redoutable.
- **Concrétude :** et les caries causent des infections.

Idiome 8

« Malgré sa déception, elle a avalé la pilule... »

- **Figuratif :** et elle a accepté les faits.
- **Littérale :** Pour se remettre de son arthrite.
- **Concrétude :** et les comprimés sont de couleur rose.

Idiome 9

« Carl est prêt à mettre la main au feu... »

- **Figuratif :** pour prouver qu’il est certain de ce qu’il affirme.
- **Littérale :** car c’est quelqu’un de maladroit**.**
- **Concrétude :** et les flammes montent au toit.

Idiome 10

« Marc n’a pas payé son café, il a laissé une ardoise... »

- **Figuratif :** et le serveur a effacé son dû.
- **Littérale :** et le couvreur a réparé le grenier.
- **Concrétude :** et la craie trace des lignes blanches.

Les idiomes non-ambigus

Idiome 1

« Son discours persuasif a réussi à lui bourrer le crâne… »

- **Figuratif :** et il a été convaincu.
- **Littérale :** et maintenant il est très lourd.
- **Concrétude :** et les os sont d’un blanc éclatant.

Idiome 2

« Après sa rupture, il passait ses journées à broyer du noir… »

- **Figuratif :** et il semblait vraiment désespéré.
- **Littérale :** et il n’en restait que de la poudre.
- **Concrétude** : et c’est un ton très sombre.

Idiome 3

« Pour réussir plus rapidement, il a brûlé les étapes… »

- **Figuratif :** mais sa démarche était trop précipitée.
- **Littérale :** et des escaliers, il n’en restait plus rien.
- **Concrétude :** et les incendies sont des catastrophes terribles.

Idiome 4

« Pendant les vacances, il aime se la couler douce… »

- **Figuratif :** en profitant de chaque moment de détente.
- **Littérale :** mais il a rapidement perdu toute sa consistance.
- **Concrétude :** et le liquide se répand rapidement.

Idiome 5

« Dans le jeu des devinettes, elle a dû donner sa langue au chat... »

- **Figuratif :** et elle a abandonné la partie.
- **Littérale :** mais il n’en a pas voulu.
- **Concrétude :** et les chiens sont très amicaux.

Idiome 6

« Son manager a tendance à lui demander la lune... »

- **Figuratif :** et il a des exigences irréalistes.
- **Littérale :** mais elle se situe à 384 400 km.
- **Concrétude :** et la nuit est silencieuse.

Idiome 7

« Quand elle a appris la nouvelle, elle était aux anges... »

- **Figuratif :** et elle avait la mine enchantée.
- **Littérale** : et elle a dû les quitter précipitamment.
- **Concrétude** : et les ailes sont de grande taille.

Idiome 8

« Le commentaire inapproprié de Marc a jeté un froid… »

- **Figuratif :** et a créé un malaise général.
- **Littérale** : et maintenant il est par terre.
- **Concrétude** : et la neige recouvre le paysage.

Idiome 9

« En attendant le rendez-vous, elle lit un livre pour tuer le temps… »

- **Figuratif :** et elle est occupée toute la matinée.
- **Littérale** : et elle trouve une bonne méthode d’exécution.
- **Concrétude** : et les meurtres sont fréquents.

Idiome 10

« Face à tant de stress, il a fini par perdre la tête… »

- **Figuratif** :  et il s’est affolé de façon ingérable.
- **Littérale :**  et maintenant il ne la localise plus.
- **Concrétude :** et le cerveau est une machine fascinante.

1. This task was presented only in French. We recommend using only the French version, as idioms are lexicalized expressions in French and not in English.
2. Pour utilisation, citer (ref de cet article)
