# Supplementary material for: Interpretation of ambiguous and unambiguous idiomatic statements in high and low schizotypal individuals
Source: Schizophr Res Cogn. 2026 Aug 21;46:100461. doi: 10.1016/j.scog.2026.100461 (PMC13524631; doi:10.1016/j.scog.2026.100461)
Supplement: Supplementary file 2 [file mmc2.docx]

**Supplementary material**

1. **Supplementary Methods**

**Selection of ambiguous idioms**

We used the ten ambiguous French idioms from Iakimova et al. (2006), which were originally selected from the IDIOMATIC database (Denhière & Verstiggel, 1998). The database contains 650 French idiomatic expressions. The database was validated to ensure equivalent levels of familiarity and salience between figurative and literal interpretations, with a mean familiarity rating of 90% (i.e., approximately 90% of participants reported knowing the expression). All ambiguous idioms selected for the present study shared the same syntactic pattern (verb–determiner–noun).

**Selection criteria for unambiguous idioms**

Since no explicit thresholds were defined by the author, we applied the operational criteria used in previous studies by Iakimova et al. (2006) and Titone et al. (2002). Idioms were thus selected according to two main criteria: they had to be highly familiar (knowledge score ≥ 26/30, approximately 87%), consistent with the familiarity scores reported by Iakimova (*M*= 90%, *SD*= 4); and they had to display low to moderate literal plausibility, with mean ratings between 1.5 and 2.25 on a 6-point scale (i.e., .10–.25 on a normalized scale). This range closely corresponds to the literally implausible idioms reported by Titone et al. (2002), whose mean literal plausibility was 2.1/7 (± .18 normalized).

**Construction of response alternatives for unambiguous idioms**

As no response options were provided in the literature, we developed our own three types of responses for each idiom. Figurative responses were based on the conventional idiomatic meaning of each expression. For example, based on the definition of the expression “bourrer le crâne”, we selected the word “convince” as the figurative option. The literal interpretation was derived by decomposing the expression into its lexical components and combining their primary dictionary meanings, yielding the literal meaning “to crush or grind something dark,” which was then summarized as *poudre* (“powder”). Concrete responses were constructed using French associative norms (Ferrand, 2001; Thérouanne & Denhière, 2004), selecting the word that was semantically or associatively the most related to the idiom’s central element.

2. **Supplementary Results**

The following supplementary analyses were conducted to further examine the potential influence of demographic variables (age and gender) and the multidimensional structure of schizotypy on idiom comprehension. These analyses were exploratory and were not part of the primary hypotheses or the main mixed-design ANOVA presented in the manuscript.

| **Table S1**  *Linear regressions examining the effect of age on idiom interpretation within each SPQ group.* | | | | | | |
| --- | --- | --- | --- | --- | --- | --- |
| **SPQ group** | **Outcome** | ***B*** | ***SE*** | ***β*** | **95% CI** | ***p*** |
| Lo-S | Figurative | .029 | .022 | .157 | [-.014, .073] | .186 |
|  | Literal | -.033 | .017 | -.225 | [-.067, .001] | .056 |
|  | Concrete | .004 | .007 | .064 | [-.010, .017] | .588 |
| Hi-S | Figurative | .020 | .030 | .084 | [-.039, .079] | .504 |
|  | Literal | -.025 | .018 | -.165 | [-.062, .012] | .186 |
|  | Concrete | .005 | .014 | .041 | [-.024, .034] | .746 |

***Note.*** *B* = unstandardized regression coefficient; *SE* = standard error; *β* = standardized regression coefficient; CI = confidence interval.

| **Table S2**  *Linear regressions examining the effect of gender on idiom interpretation within each SPQ group.* | | | | | | |
| --- | --- | --- | --- | --- | --- | --- |
| **SPQ group** | **Outcome** | ***B*** | ***SE*** | ***β*** | **95% CI** | ***p*** |
| Lo-S | Figurative | -1.789 | .814 | -.252 | [-3.411, -.166] | .031 |
|  | Literal | 1.378 | .637 | .249 | [.108, 2.647] | .034 |
|  | Concrete | .411 | .248 | .193 | [-.083, 0.905] | .101 |
| Hi-S | Figurative | -1.019 | 1.038 | -.123 | [-3.093, 1.054] | .330 |
|  | Literal | .712 | .676 | .131 | [-.640, 2.063] | .297 |
|  | Concrete | .308 | .496 | .078 | [-.683, 1.299] | .537 |

***Note.*** *B*= unstandardized regression coefficient; *SE*= standard error; *β*= standardized regression coefficient; CI= confidence interval. Gender was coded as 1= female and 2= male.

| **Table S3**  *Multiple linear regression analyses examining the unique contributions of the three SPQ dimensions to response type (N = 377).* | | | | | | | | |
| --- | --- | --- | --- | --- | --- | --- | --- | --- |
| **Outcome** | **Predictor** | **Estimate** | ***SE*** | ***β*** | ***t*** | ***p*** | **95% CI LL** | **95% CI UL** |
| Figurative responses | Cognitive-perceptual | .007 | .044 | .010 | .154 | .878 | -.080 | .094 |
|  | Interpersonal | .011 | .034 | .018 | .317 | .751 | -.057 | .078 |
|  | Disorganization | -.177 | .060 | -.202 | -2.955 | .003 | -.295 | -.059 |
| Literal responses | Cognitive-perceptual | -.019 | .031 | -.039 | -.598 | .550 | -.081 | .043 |
|  | Interpersonal | .011 | .024 | .027 | .467 | .641 | -.037 | .059 |
|  | Disorganization | .103 | .043 | .167 | 2.425 | .016 | .020 | .187 |
| Concrete responses | Cognitive-perceptual | .015 | .017 | .057 | .889 | .374 | -.018 | .049 |
|  | Interpersonal | -.018 | .013 | -.075 | -1.318 | .188 | -.044 | .009 |
|  | Disorganization | .066 | .023 | .193 | 2.830 | .005 | .020 | .112 |

***Note.*** *Estimate*= unstandardized regression coefficient; *SE*= standard error; *β*= standardized regression coefficient; CI= confidence interval. The three SPQ dimensions were entered simultaneously into each regression model.

**Conclusion**

Overall, although several regression coefficients reached statistical significance, the corresponding standardized effects were small (|β| ≤ .25) and should therefore be interpreted with caution.
